# Impact of the COVID-19 pandemic on changes in temperature-sensitive cardiovascular and respiratory disease mortality in Japan

**DOI:** 10.1371/journal.pone.0275935

**Published:** 2022-10-10

**Authors:** Yukitaka Ohashi, Yuya Takane, Ko Nakajima

**Affiliations:** 1 Faculty of Biosphere-Geosphere Science, Okayama University of Science, Okayama City, Japan; 2 Environmental Management Research Institute, National Institute of Advanced Industrial Science and Technology (AIST), Tsukuba City, Ibaraki, Japan; The Chinese University of Hong Kong, HONG KONG

## Abstract

Some cardiovascular and respiratory diseases are triggered by changes in ambient temperature or extremes of temperature. This study aimed to clarify the changes in mortality associated with temperature-sensitive diseases in Japan during the COVID-19 pandemic. We used data from three major cities (Sapporo City, Tokyo 23 wards, and Osaka City) from 2010 to 2019 to determine disease mortality rates and monthly mean temperatures from April to December. If the pandemic had not occurred in 2020, the results showed that temperature-sensitive disease death counts would have increased from 324 to 980, based on a 95% confidence interval estimated from the past 10 years in Sapporo (19–56% increase in actual deaths from 2020), from 651 to 2,653 in Tokyo (10–39% increase), and from 235 to 1,343 in Osaka (8–48% increase). Analyses of meshed population data during the COVID-19 pandemic indicated that inhibiting people’s behaviour and outdoor mobility, especially in older men, caused a decrease in mortality.

## Introduction

In 2020, the novel coronavirus disease 2019 (COVID-19) pandemic, caused by the severe acute respiratory syndrome coronavirus 2, disrupted daily life worldwide. Lockdown policies strongly inhibited outdoor activities in many countries (e.g., France, Germany, Italy, the United Kingdom, Ghana, China, India, Australia, the United States, and Argentina). Also, in Japan, people were often required to change facets of their daily lives, such as work and school, to “stay at home” due to the repeated COVID-19 state-of-emergency declarations from the Japanese government [1]. This behaviour modification marked decreases in other infectious diseases, such as seasonal influenza and respiratory syncytial virus [2]. In addition, there were very few excess all-cause deaths during the COVID-19 pandemic (e.g., only 0.03–0.72% of deaths were excess deaths reported at the national level from January to May 2020 [3]), whereas diseases in noncommunicable disease (such as cardiovascular disease) and pneumonia (excluding COVID-19) deaths were reported by the Japanese Ministry of Health, Labour and Welfare (MHLW) [4]. The number of deaths due to cardiovascular and respiratory diseases in 2020 during the pandemic was 345,476 and 172,727, respectively. This was a significant reduction of 5,029 and 20,507 compared to 2019 (pre-COVID-19), respectively.

Therefore, analysing the changes in the number of cardiovascular and respiratory deaths induced by behavioural modifications will provide important information for wide-ranging issues, such as public health, climate change, and the ageing population. The occurrence of cardiovascular and respiratory diseases that are strongly related to ambient temperature is expected to be affected by changes in daily personal activities and lifestyle behaviours [5]. Modifications in people’s daily behaviours most likely altered disease risk conferred by the ambient temperature patterns during the COVID-19 pandemic.

Epidemiological studies in many countries, including Japan, have shown that cardiovascular and respiratory diseases are vulnerable to changes in the weather and climate [6–9]. In particular, ambient temperature can be related to morbidity or mortality, and both cold and hot exposure may increase the risk of developing cardiovascular and respiratory diseases [10–15]. Several meta-analyses revealed an increase of 5% (cold) and 1.3% (hot) in cardiovascular mortality risk from studies from 2000–2015 [6] and an increase of 1.4% and 2.9% in cerebrovascular and respiratory mortality, respectively, with a temperature rise of 1°C in hot conditions [16].

On 10 September 2021, the Japanese government published the final statistical data on deaths in 2020. As aforementioned, it should be possible to estimate from the temperature measured in 2020 what the number of deaths due to cardiovascular and respiratory diseases would have been if no pandemic had occurred in 2020. Therefore, this study aimed to clarify the effects of human activity restrictions on the risk of death from these temperature-sensitive diseases. Considering the connection between human behaviour, ambient temperature and disease mortality, the findings are expected to be novel and provide a new understanding of these diseases.

## Data and methods

### Study area

**Fig 1:**
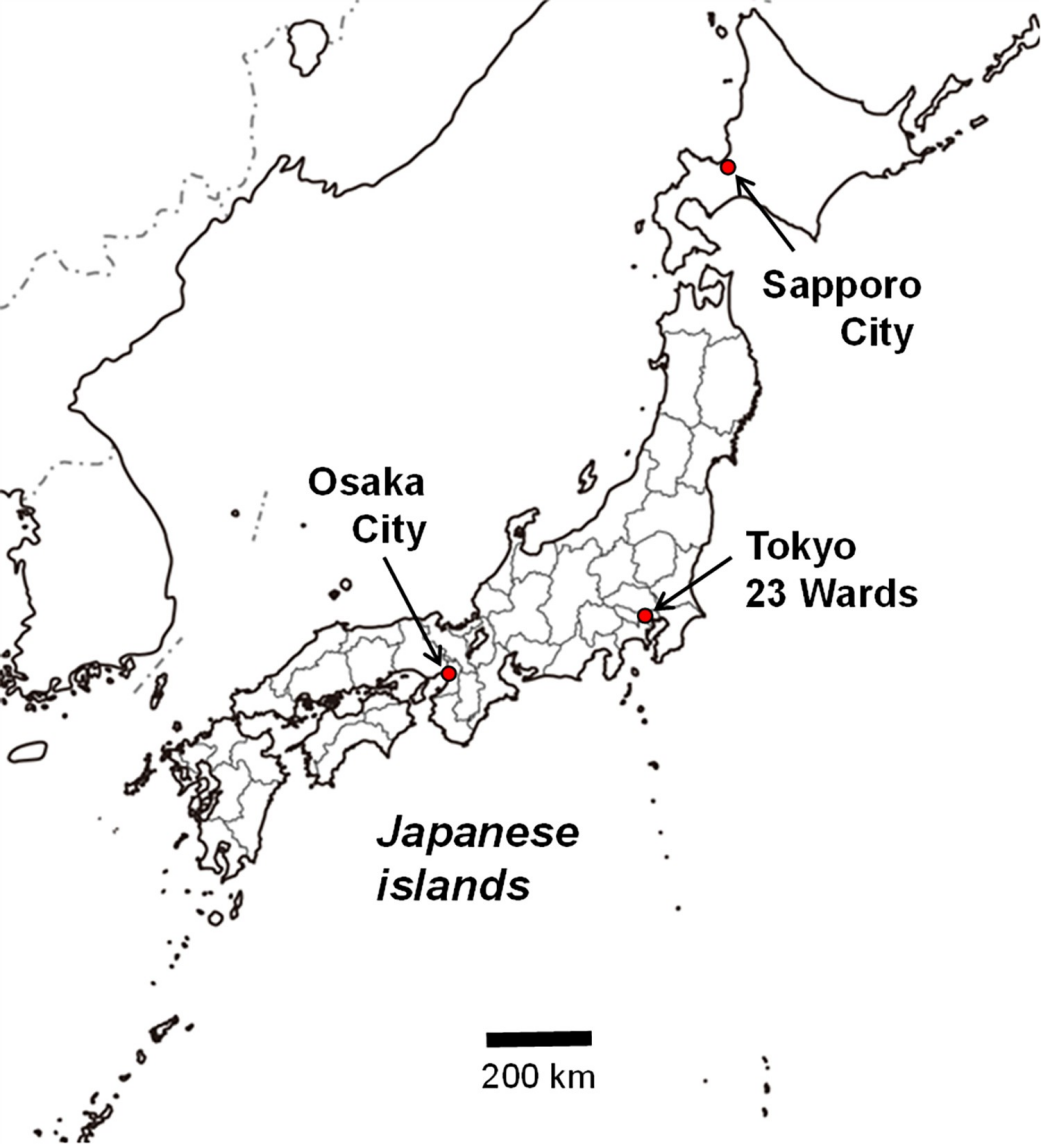
Locations of Sapporo City, Tokyo 23 wards, and Osaka City in Japan. The map is provided from https://www.freemap.jp/ licensed under CC BY 4.0.

This study targeted three major cities (Fig 1): Sapporo City (hereinafter Sapporo), Tokyo 23 wards (Tokyo), and Osaka City (Osaka), with a population of approximately 1.97 million, 9.71 million, and 2.75 million in 2021, respectively. These cities are among the top five most populous cities in Japan. We also analysed these cities because there are large differences in climate conditions between each city; the monthly mean ambient temperatures (the average from 1991–2020) [17] were 22.3°C in Sapporo, 26.9°C in Tokyo, and 29.0°C in Osaka during the summer month of August, while mean temperatures during the winter month of December were **−**0.9°C in Sapporo, 15.8°C in Tokyo, and 17.1°C in Osaka. This comparison is expected to reveal the influence of domestic climate differences on mortality changes in temperature-sensitive cardiovascular and respiratory diseases if the COVID-19 pandemic had not occurred in Japan.

### Death data

On the Japanese government’s portal site “e-Stat”, the monthly number of deaths is published for each cause of death (available online at https://www.e-stat.go.jp). The data are highly reliable because of the statistical reports by the MHLW organised by the Japanese government. In this study, the following seven diseases were analysed: ischaemic heart disease (IHD), including acute myocardial infarction, cardiac arrhythmia and conduction disorders (CACD), heart failure (HF), intracerebral haemorrhage (ICH), cerebral infarction (CI), and respiratory diseases (Resp). IHD, CACD, HF, ICH, CI, and Resp correspond to the sum of I20–25 (classification code), I44–49, I50, I61 (including I69.1), I63 (including I69.3), and J00–99, respectively, based on the International Statistical Classification of Diseases and Related Health Problems, 10th revision (ICD-10) [18]. These diseases are known to be temperature-sensitive, as shown by previous studies [6, 7, 10, 13, 16, 19–21]. However, subarachnoid haemorrhage, a representative cerebrovascular disease, was excluded from our analyses because of the low sensitivity of mortality to air temperature and season in Japan [22]. Moreover, the number of deaths due to this disease was smaller than that of other diseases, corresponding to approximately 1–2% of IHD and half of CACD.

To eliminate yearly changes and city differences in the population by age group, the age-adjusted mortality rate (*MR*_adj_) was used.


$${MR}_{adj}=\frac{\sum_{k}\left({{MR}_{k}∙P}_{k}\right)}{\sum_{k}P_{k}}$$ (1)

Where *k* is the age group number separated into five years. *MR*_k_ and *P*_k_ in Eq (1) correspond to the mortality rate and standard population for a specific age group, *k*, respectively. The population structure by age group in 2015 was used for*P*_k_. The *MR*_adj_ for each disease was calculated per 100,000 (for older people aged ≥65) in each city. In Japan, the number of deaths due to cardiovascular and respiratory diseases was almost wholly comprised of people aged ≥65; in particular, the HF, CI, and Resp deaths in Sapporo City, Tokyo’s 23 wards, and Osaka City were more than 95% from 2018 to 2020. Therefore, death data from older individuals aged ≥65 were used in this study.

### Temperature data

The monthly mean temperature (*MMT*) was obtained from the open data observed and reported by the Japan Meteorological Agency (JMA) (available online at https://www.jma.go.jp). Many weather stations, called the Automated Meteorological Data Acquisition System (AMeDAS), have been placed by the JMA throughout Japan. High-quality meteorological data measured by the JMA have been used in numerous studies in a wide range of academic fields over a long period. The respective AMeDAS within Sapporo City, Ota Ward in Tokyo (23 wards), and Osaka City were chosen for the analyses. The outdoor air temperature was measured 1.5 m above the ground, which was forced-ventilated using a radiation shield.

Spatial representation of the observed temperature was a problem because only one site was used in each city. However, the absolute air temperature values were less important for our analyses than the relative annual variations in the MMT.

### Population data

To understand spatio-temporal activities, the mobile spatial statistics (MSS) data in Sapporo, Tokyo, and Osaka retrieved by Nippon Telegraph and Telephone (NTT) Docomo, Inc. (Tokyo, Japan) were analysed in this study. The MSS data provides population statistics based on the location information of 78 million users (15–79 years old) of NTT Docomo’s mobile terminals. Detailed estimation methods for MSS have been described by Terada et al. [23], Nakajima et al. [24], and Takane et al. [25]: (1) the number of mobile terminals in each base station area is aggregated; (2) the total number of mobile terminals is extrapolated using the adoption rates of NTT Docomo mobile terminals; and (3) the estimated population is re-aggregated into each grid section. Then, the 500-m mesh data (aggregated as 1 km mesh in this study) were produced hourly by age group every 10 years. Thus, MSS provides information that helps examine real-time changes in specific populations.

In addition, Google COVID-19 community mobility reports (available online at https://www.google.com/covid19/mobility/) were used. These open data were analysed from the location information acquired by mobile terminals worldwide and summarised as a mobility change rate based on the pre-COVID-19 period (1 January–6 February 2020) in each prefecture or state in each county. Furthermore, the daily change rates were indicated in different categories of places, such as “retail and recreation”, “groceries and pharmacies”, “parks”, “transit stations”, “workplaces”, and “residential”.

### Analysis periods

**Fig 2:**
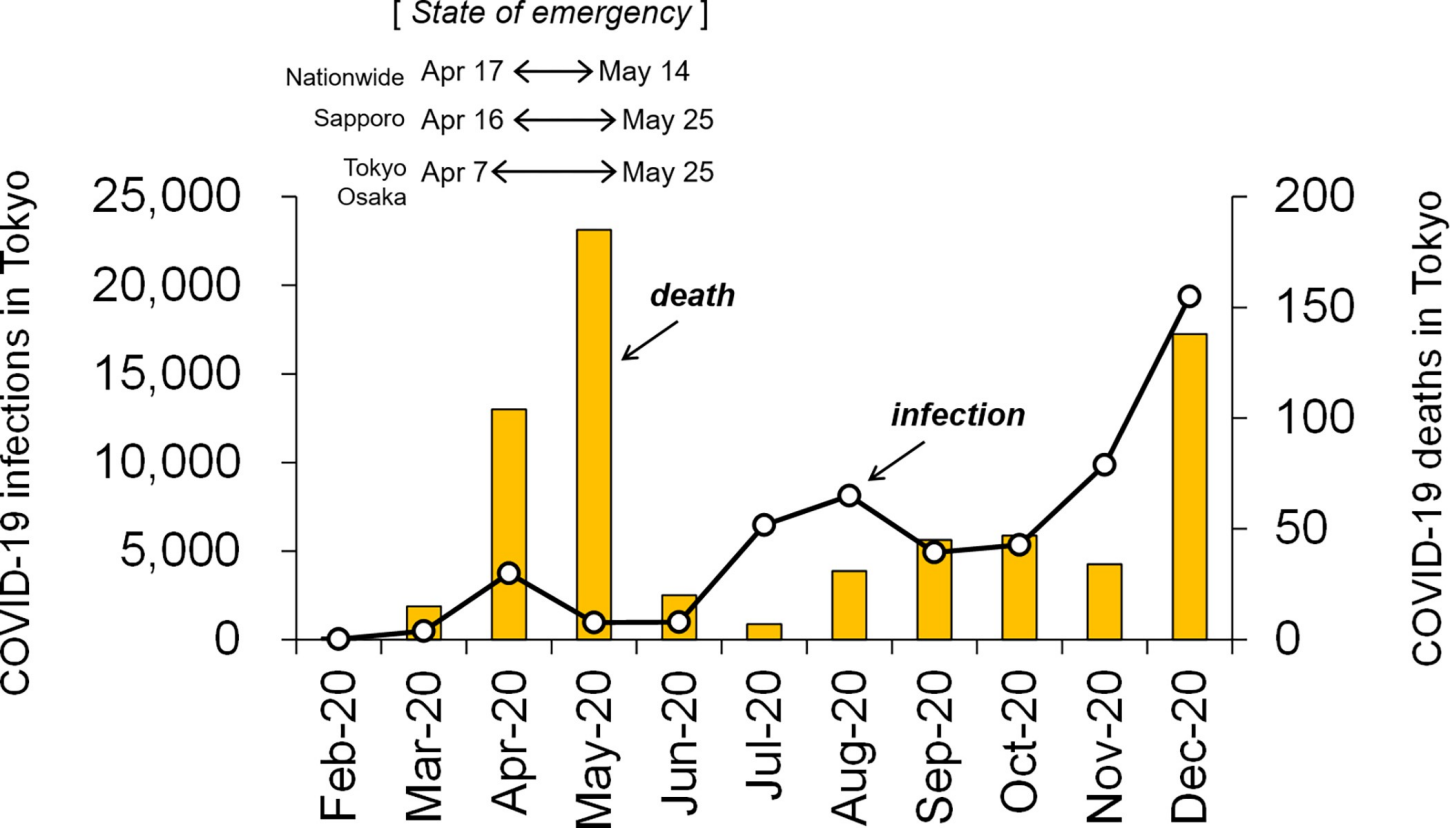
Monthly variations of COVID-19 infections and deaths in Tokyo 23 wards from February to December 2020. The Japanese government declared a state of emergency restricting people’s daily activities from April 7 (Tokyo and Osaka) and 16 (Sapporo) to May 25 in 2020.

The difference between the pre-COVID-19 and pandemic societies in Japan was focused on extracting the impact of the COVID-19 spread on human cardiovascular and respiratory disease deaths in response to ambient temperatures. As COVID-19 infections rapidly increased in March and April, 2020 (Fig 2), the Japanese government declared a state of emergency and limited daily activities from 7 April (Tokyo and Osaka) or 16 April (Sapporo) to 25 May 2020. Because a nationwide emergency was declared from 17 April to 14 May 2020, the population mesh data were analysed for this period. In this study, the months after March (to December) in 2020 were analysed as the COVID-19 pandemic period, while the months from 2010 to 2019 were compared with those of the pre-pandemic normal period. Because the final death data for 2021 have not yet been published on the Japanese government portal site, the 2020 death data used in this study are the latest information on the COVID-19 pandemic.

## Results

### Mortality response to temperature

**Fig 3:**
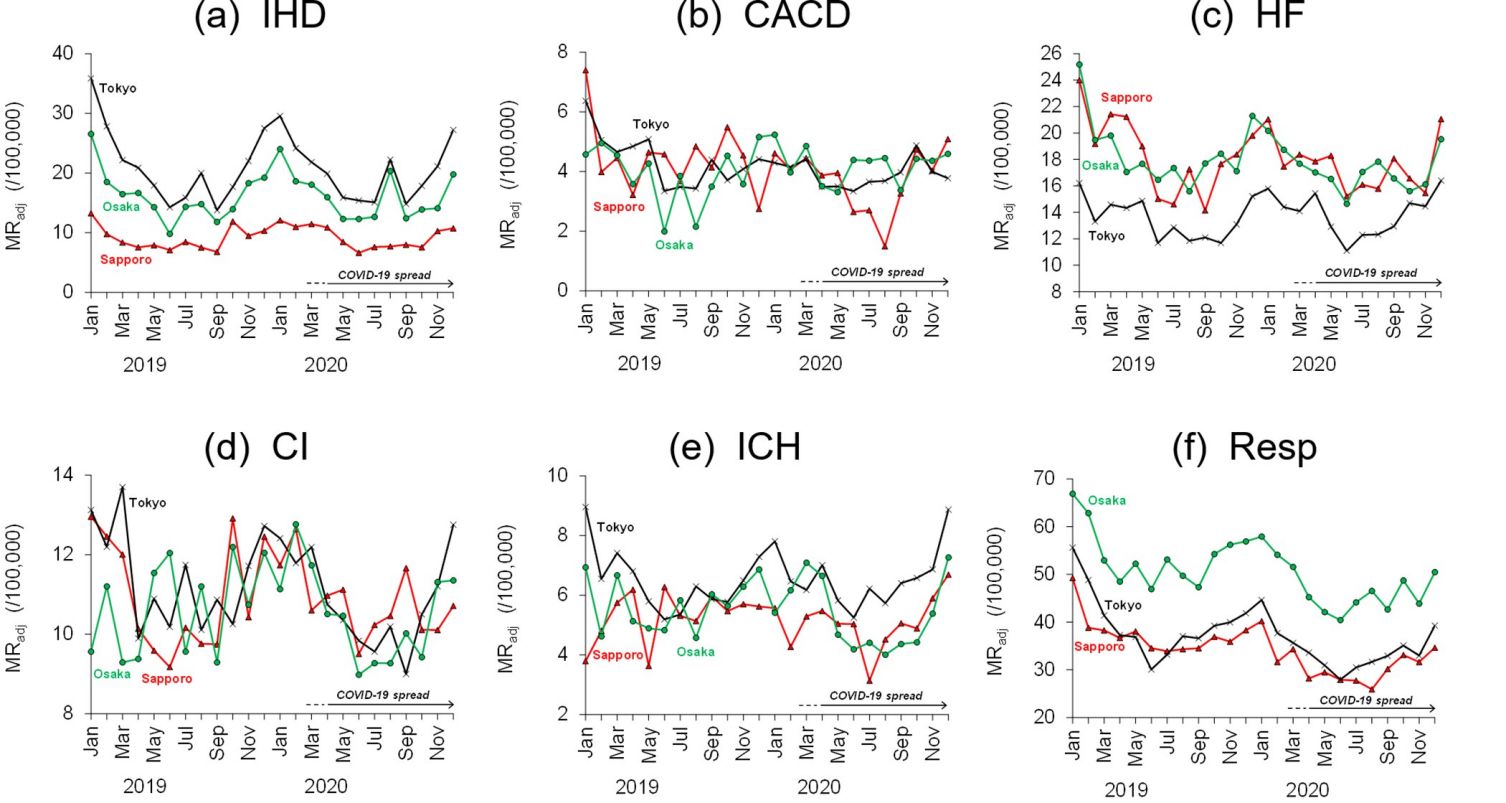
Monthly variations of each *MR*_adj_ from 2019 to 2020. Mortality rates of (a) acute ischaemic heart disease (IHD), (b) cardiac arrhythmia and conduction disorder (CACD), (c) heart failure (HF), (d) cerebral infarction (CI), (e) intracerebral haemorrhage (ICH), and (f) respiratory diseases (Resp) are shown for Sapporo, Tokyo, and Osaka. In Japan, COVID-19 began to spread in March 2020 (cf. Fig 2).

The *MR*_adj_ from January 2019 to December 2020 for IHD, CACD, HF, CI, ICH, and Resp gradually increased in winter and decreased in summer (Fig 3). Many studies have reported cardiovascular and respiratory mortality increases in older people during the cold months [6, 10, 15, 16, 26–28]. In Japan, COVID-19 began to spread in March 2020 (cf. Fig 2) [29]. Because decreases in *MR*_adj_ from spring to summer were found not only in 2020 but also in 2019 (pre-COVID-19 period), the overlap between the decreasing period and the COVID-19 spreading period seems to be incidental.

Ohashi et al. [22] revealed yearly *MR*_adj_ responses for these diseases to the monthly mean temperature (*MMT*). A linear regression analysis was also performed to explain the response of *MR*_adj_ to *MMT* in 2010–2019 of the pre-COVID-19 years and to obtain the regression coefficient for each disease (Table 1). Statistical validation in the use of linear regression was verified by the Breusch–Pagan test [30], which detected heteroscedasticity of the residual term for all diseases and months excluding HF in October in Osaka.

**Table 1:** Regression results of *MR*_adj_ to *MMT* obtained from the pre-COVID-19 pandemic years (2010–2019). The results of acute ischemic heart disease (IHD), cerebral infarction (CI), and respiratory diseases (Resp) in each month. Parentheses indicate the *p*-value with a t-test for a regression coefficient at the 90%, 95%, and 99% levels, respectively.

Regression coefficient	**January**			**February**			**March**		
	Sapporo	Tokyo	Osaka	Sapporo	Tokyo	Osaka	Sapporo	Tokyo	Osaka
IHD	+0.41 (0.579)	−1.75 (0.203)	−2.63 (0.145)	−0.19 (0.732)	−2.22 (0.032)	−1.28 (0.215)	−1.58 (0.017)	−2.01 (0.030)	−1.98 (0.027)
CI	+0.24 (0.810)	−2.45 (0.180)	−3.04 (0.114)	−0.01 (0.988)	−1.33 (0.396)	+0.21 (0.879)	−2.44 (0.005)	−1.95 (0.014)	−2.23 (0.107)
Resp	−1.96 (0.415)	−4.06 (0.146)	−4.92 (0.238)	−2.19 (0.328)	−3.84 (0.059)	−1.42 (0.334)	−1.72 (0.167)	−1.82 (0.201)	−1.66 (0.453)
Regression coefficient	**April**			**May**			**June**		
	Sapporo	Tokyo	Osaka	Sapporo	Tokyo	Osaka	Sapporo	Tokyo	Osaka
IHD	−1.95 (0.044)	−2.11 (0.003)	−1.95 (0.007)	−1.39 (0.024)	−1.58 (0.003)	−2.61 (0.011)	+1.09 (0.160)	+0.35 (0.819)	+2.46 (0.253)
CI	−1.93 (0.024)	−1.69 (0.072)	−2.07 (0.074)	−1.11 (0.066)	−3.83 (0.001)	−3,97 (<0.001)	+2.31 (0.017)	−0.61 (0.782)	+2.34 (0.291)
Resp	−2.25 (0.151)	−2.78 (0.071)	−4.17 (0.066)	−3.01 (0.051)	−4.96 (0.019)	−5.27 (0.011)	+6.13 (0.020)	−1.16 (0.798)	+5.69 (0.266)
Regression coefficient	**July**			**August**			**September**		
	Sapporo	Tokyo	Osaka	Sapporo	Tokyo	Osaka	Sapporo	Tokyo	Osaka
IHD	+1.49 (0.189)	+1.98 (0.085)	+0.78 (0.503)	+1.07 (0.218)	+1.42 (0.131)	+1.46 (0.333)	+0.23 (0.697)	+0.11 (0.893)	+0.56 (0.351)
CI	+1.13 (0.455)	+0.47 (0.658)	−1.11 (0.543)	+1.18 (0.167)	+0.59 (0.641)	+1.79 (0.236)	+0.64 (0.591)	+1.31 (0.225)	+0.25 (0.768)
Resp	+0.40 (0.897)	+1.12 (0.530)	−3.41 (0.275)	+3.94 (0.016)	+0.83 (0.700)	+2.29 (0.422)	+1.05 (0.583)	+0.61 (0.715)	−1.29 (0.291)
Regression coefficient	**October**			**November**			**December**		
	Sapporo	Tokyo	Osaka	Sapporo	Tokyo	Osaka	Sapporo	Tokyo	Osaka
IHD	+0.65 (0.197)	−1.34 (0.356)	−0.61 (0.649)	−0.11 (0.717)	−1.81 (0.094)	−1.19 (0.288)	+0.38 (0.641)	−1.62 (0.087)	−2.10 (0.084)
CI	+0.53 (0.614)	−1.49 (0.296)	−0.23 (0.873)	+1.38 (0.069)	−0.60 (0.652)	−1.13 (0.269)	+0.17 (0.867)	−1.26 (0.237)	−0.98 (0.168)
Resp	−2.71 (0.298)	−1.74 (0.554)	+0.95 (0.760)	+1.02 (0.439)	−2.52 (0.309)	−1.86 (0.439)	−0.75 (0.776)	−2.24 (0.323)	−1.11 (0.682)

**Fig 4:**
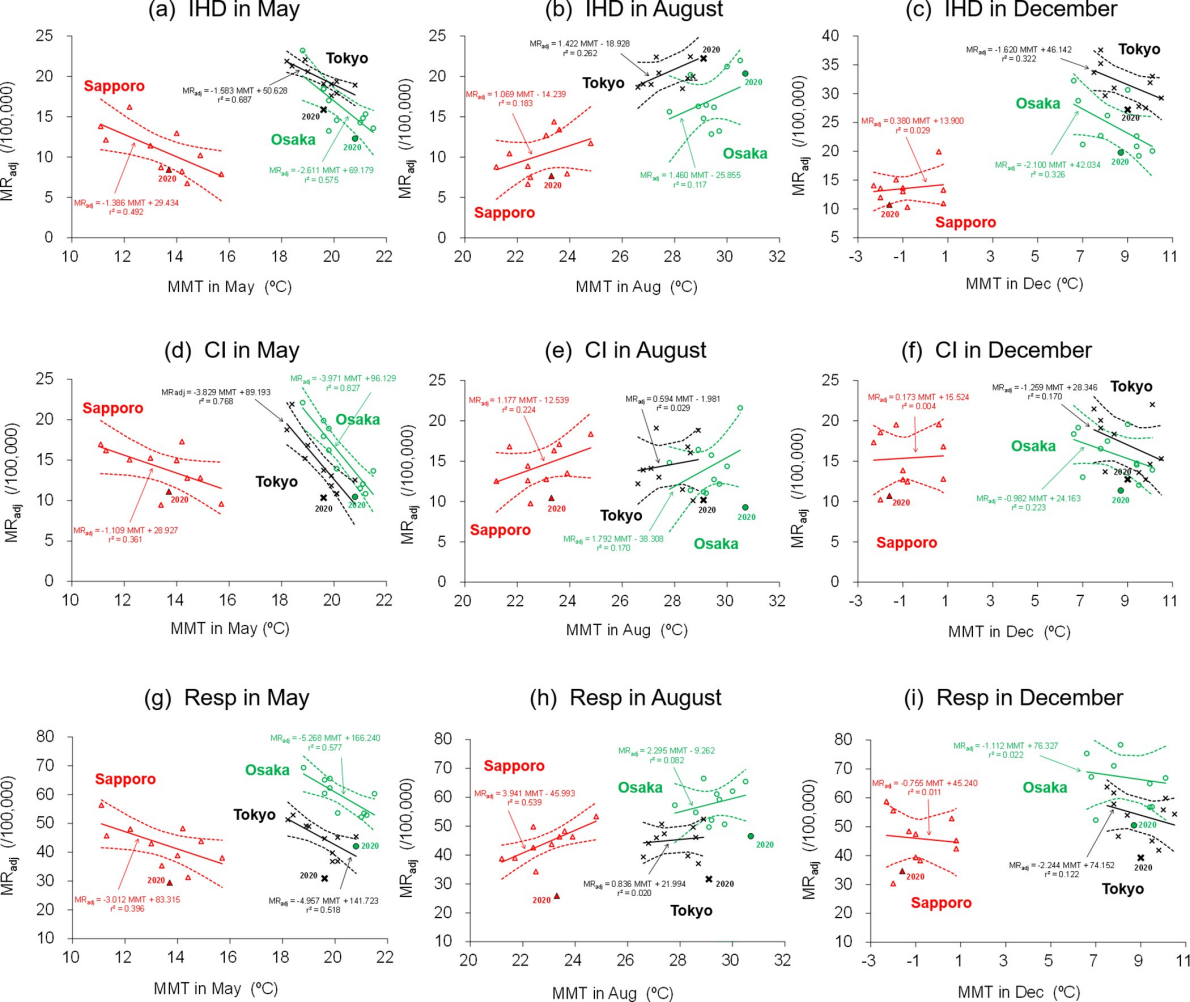
The *MR*_adj_ sensitivity to *MMT* analysed from the past data and the 2020 plots in Sapporo (red triangle), Tokyo (black cross), and Osaka (green circle). The results of (a, b, c) acute ischemic heart disease (IHD), (d, e, f) cerebral infarction (CI), and (g, h, i) respiratory diseases (Resp) are exhibited for May, August, and December. Straight regression line and the 95% confidence interval (broken curve line) are presented for the pre-pandemic period (2010–2019), while filled marks represent the results of the COVID-19 pandemic year (2020). Regression equations and their contribution rates are also shown for the regression lines.

As an example, the linear responses of the IHD, CI, and Resp *MR*_adj_ to *MMT* in May, August, and December, which were determined from 2010 to 2019, are shown in Fig 4 (the other diseases are displayed in S1 Fig). In May (Fig 4A, 4D and 4G), *MR*_adj_ increased with decreasing annual *MMT* in all cities, with statistically significant correlations for May IHD in Sapporo (*r* = 0.70; *p*<0.05), Tokyo (*r* = 0.83; *p*<0.01), and Osaka (*r* = 0.76; *p*<0.05); May CI in Tokyo (*r* = 0.88; *p*<0.01) and Osaka (*r* = 0.91; *p*<0.01); May Resp in Tokyo (*r* = 0.74; *p*<0.05) and Osaka (*r* = 0.76; *p*<0.05). Hence, the *MR*_adj_ in May can be regarded as having a high contribution rate (*r*^2^) to the *MMT* for most diseases in the three cities.

In May Tokyo and Osaka showed a significant negative response of *MR*_adj_ to *MMT*, despite having temperate climate conditions with a higher *MMT* than Sapporo. This difference is probably attributed to the fact that the “optimum temperature” at which temperature-sensitive mortality is the lowest during the year [31, 32] differs by region. In addition, the August *MR*_adj_ of any city (Fig 4B, 4E and 4H) showed a positive response to *MMT*, even in Sapporo, where the August *MMT* was significantly lower than that in Tokyo and Osaka. Hence, disease mortality tends to increase if the temperature in a given year is higher or lower than the climatologically optimum temperature of a city [21, 31, 32].

In December (Fig 4C, 4F and 4I), negative responses of the disease *MR*_adj_ to MMT appeared similar to May, excluding Sapporo, with the response unclear. The abovementioned monthly response characteristics indicate whether extraordinary *MR*_adj_ was observed against the measured *MMT* in 2020 during the COVID-19 pandemic.

### Mortality decrease in 2020

The results of *MR*_adj_ and *MMT* observed in 2020 during the COVID-19 spread were overlaid on those of the pre-COVID-19 period (Fig 4). In particular, the *MR*_adj_ of IHD in Tokyo in May 2020 (Fig 4A), CI in Sapporo and Osaka in August 2020 (Fig 4E), CI in Osaka in December 2020 (Fig 4F), and Resp in all cities and months (Fig 4G–4I) were lower than the lower end, showing an estimated range with a 95% confidence interval for a linear regression line determined from the past 10 years (pre-COVID-19). If COVID-19 has not occurred, the value of *MR*_adj_ should remain within this range in response to the 2020 *MMT*. The CI and Resp *MR*_adj_ in Tokyo and Osaka showed the largest decrease since 2010, although the August *MMT* in 2020 was observed to have the highest value since 2010 in both cities. Therefore, these data in 2020 can be judged as unusual decreases, which would have been in the 95% confidence interval range for the past 10 years if Japanese society had experienced a normal 2020 year without the COVID-19 pandemic.

From the above point of view, in terms of IHD and CI (Tables 2 and 3), the influence of the COVID-19 pandemic on the *MR*_adj_ of each disease was quantitated, including the pre-COVID-19 and spreading COVID-19 months (January–March) in 2020. In the tables, “*MMT* contribution rate from 10 years” and “expected natural *MR*_adj_” correspond to the contribution rate (*r*^2^) and the expected value of *MR*_adj_ if not for the pandemic, respectively, which were estimated from the regression line in Fig 4. For example, although the IHD *MR*_adj_ expected from 13.4°C (lower than the 10-years average of 14.5°C) of *MMT* in April 2020 was 25.47 in Tokyo, it was recorded as 19.87 (Table 2). This means that the IHD *MR*_adj_ decreased by 22.0% (“predictable change of *MR*_adj_” in the table) from the anticipated value under the influence of the COVID-19 pandemic, which was lower than those in the past 10 unaffected years (shown as “Yes” in “statistical confidence” in the tables when outside the 95% confidence interval for linear regression).

**Table 2:** Predictable changes in *MR*_adj_ for acute ischaemic heart disease (IHD) estimated in this study.

		**January (Pre-COVID-19 in 2020)**			**February (Pre-COVID-19 in 2020)**			**March (Spreading COVID-19 in 2020)**			
		Sapporo	Tokyo	Osaka	Sapporo	Tokyo	Osaka	Sapporo	Tokyo	Osaka	
2020 result	*MMT* (°C)	−2.3	8.2	8.6	−2.1	9.1	8.0	3.3	11.4	11.4	
	*MR*_adj_	12.05	29.56	24.00	10.98	24.16	18.61	11.43	16.42	18.05	
*MMT* contribution rate from 10 years (%)		4.0	19.4	24.5	1.6	45.6	18.4	52.7	46.2	47.5	
Expected natural *MR*_adj_		14.06	33.45	21.14	11.20	25.06	21.70	9.10	25.10	18.52	
Predictable change of *MR*_adj_ (%)		**−**14.3	**−**11.6	**+**13.5	**−**2.0	**−**3.6	**−**14.2	+25.6	**−**13.1	**−**2.5	
Statistical confidence		No	No	No	No	No	No	No	No	No	
		**April (Prevalent COVID-19 in 2020)**			**May (Prevalent COVID-19 in 2020)**			**June (Prevalent COVID-19 in 2020)**			
		Sapporo	Tokyo	Osaka	Sapporo	Tokyo	Osaka	Sapporo	Tokyo	Osaka	
2020 result	*MMT* (°C)	6.8	13.4	13.7	13.7	19.6	20.8	18.3	23.3	24.9	
	*MR*_adj_	10.87	19.87	15.93	8.44	15.87	12.29	6.62	15.39	12.28	
*MMT* contribution rate from 10 years (%)		41.7	**69.0**	**61.4**	**49.2**	**68.7**	**57.5**	**23.0**	0.7	16.0	
Expected natural *MR*_adj_		11.56	**25.47**	**21.41**	**10.45**	**19.60**	**14.87**	**11.47**	18.37	17.18	
Predictable change of *MR*_adj_ (%)		**−**6.0	**−22.0**	**−25.6**	**−19.2**	**−19.0**	**−17.4**	**−42.3**	**−**16.2	**−**28.5	
Statistical confidence		No	**Yes**	**Yes**	**Yes**	**Yes**	**Yes**	**Yes**	No	No	
		**July (Prevalent COVID-19 in 2020)**			**August (Prevalent COVID-19 in 2020)**			**September (Prevalent COVID-19 in 2020)**			
		Sapporo	Tokyo	Osaka	Sapporo	Tokyo	Osaka	Sapporo	Tokyo		Osaka
2020 result	*MMT* (°C)	21.2	24.7	26.0	23.3	29.1	30.7	20.1	24.9		25.8
	*MR*_adj_	7.59	15.08	12.65	7.68	22.23	20.35	7.98	14.82		12.41
*MMT* contribution rate from 10 years (%)		20.5	32.5	5.8	**18.3**	26.2	11.7	2.0	0.2		**10.9**
Expected natural *MR*_adj_		9.30	17.19	15.70	**10.67**	22.45	18.97	10.48	17.27		**14.81**
Predictable decrease of *MR*_adj_ (%)		−18.4	−12.3	−19.4	**−28.0**	−1.0	+7.3	**−23.9**	**−14.2**		**−16.2**
Statistical confidence		No	No	No	**Yes**	No	No	**Yes**	**Yes**		**Yes**
		**October (Prevalent COVID-19 in 2020)**			**November (Prevalent COVID-19 in 2020)**			**December (Prevalent COVID-19 in 2020)**			
		Sapporo	Tokyo	Osaka	Sapporo	Tokyo	Osaka	Sapporo	Tokyo	Osaka	
2020 result	*MMT* (°C)	13.1	18.2	18.7	6.3	15.0	14.7	−1.6	9.0	8.7	
	*MR*_adj_	7.55	17.84	13.88	10.27	21.13	14.09	10.73	27.23	19.77	
*MMT* contribution rate from 10 years (%)		**19.8**	**10.7**	2.7	1.7	31.1	**13.9**	2.9	**32.2**	**32.6**	
Expected natural *MR*_adj_		**11.49**	**21.91**	17.92	10.32	22.74	**17.78**	13.29	**32.56**	**23.76**	
Predictable change of *MR*_adj_ (%)		**−34.3**	**−18.6**	**−22.5**	−0.5	−7.1	**−20.8**	**−19.3**	**−13.7**	**−16.8**	
Statistical confidence		**Yes**	**Yes**	**Yes**	No	No	**Yes**	**Yes**	**Yes**	**Yes**	

The “predictable change of *MR*_adj_” indicates the decrease rate (%) to “expected natural *MR*_adj_” estimated using a regression line determined by the past 10 year data. The bold numerals and characters in “predictable change of *MR*_adj_” and “statistical confidence” indicate a lower value than that at 95% confidence interval determined from the past 10 years (shown as “Yes” in “statistical confidence”). In addition, the grey-coloured column corresponds to the city and month having a temperature-sensitive IHD satisfying “*MMT* contribution rate from 10 years” greater than 10%.

**Table 3:** Predictable changes in *MR*_adj_ for cerebral infarction (CI) estimated in this study.

		**January (Pre-COVID-19 in 2020)**			**February (Pre-COVID-19 in 2020)**				**March (Spreading COVID-19 in 2020)**					
		Sapporo	Tokyo	Osaka	Sapporo	Tokyo	Osaka		Sapporo		Tokyo		Osaka	
2020 result	*MMT* (°C)	−2.3	8.2	8.6	−2.1	9.1	8.0		3.3		11.4		11.4	
	MR_adj_	11.73	12.41	11.13	12.65	11.79	12.76		10.60		12.19		11.73	
*MMT* contribution rate from 10 years (%)		0.8	21.2	28.2	0.0	9.1	0.3		64.7		55.1		29.2	
Expected natural *MR*_adj_		12.48	13.70	8.52	14.54	13.29	16.82		10.27		14.52		13.53	
Predictable change of *MR*_adj_ (%)		**−6.0**	−9.4	+30.7	−13.0	−11.3	−24.1		+3.2		−16.0		−13.3	
Statistical confidence		**Yes**	No	No	No	No	No		No		No		No	
		**April (Prevalent COVID in 2020)**			**May (Prevalent COVID in 2020)**					**June (Prevalent COVID in 2020)**				
		Sapporo	Tokyo	Osaka	Sapporo	Tokyo		Osaka		Sapporo		Tokyo		Osaka
2020 result	*MMT* (°C)	6.8	13.4	13.7	13.7	19.6		20.8		18.3		23.3		24.9
	*MR*_adj_	10.97	10.75	10.51	11.12	10.35		10.46		9.50		9.83		8.98
*MMT* contribution rate from 10 years (%)		49.0	34.9	34.6	36.1	76.8		82.7		52.9		1.0		13.8
Expected natural *MR*_adj_		13.91	16.87	19.23	13.73	14.14		13.53		15.17		12.58		17.15
Predictable decrease of *MR*_adj_ (%)		**−21.1**	**−36.3**	**−45.3**	**−19.0**	**−26.8**		**−22.7**		**−37.4**		−21.9		**−47.7**
Statistical confidence		**Yes**	**Yes**	**Yes**	**Yes**	**Yes**		**Yes**		**Yes**		No		**Yes**
		**July (Prevalent COVID in 2020)**			**August (Prevalent COVID in 2020)**				**September (Prevalent COVID in 2020)**					
		Sapporo	Tokyo	Osaka	Sapporo	Tokyo	Osaka		Sapporo		Tokyo			Osaka
2020 result	*MMT* (°C)	21.2	24.7	26.0	23.3	29.1	30.7		20.1		24.9			25.8
	*MR*_adj_	10.23	9.56	9.27	10.46	10.19	9.27		11.66		9.00			10.02
*MMT* contribution rate from 10 years (%)		7.2	2.6	4.8	22.4	2.9	17.0		3.8		17.8			1.1
Expected natural *MR*_adj_		13.52	13.62	16.50	15.05	15.30	16.71		13.89		15.08			13.89
Predictable decrease of *MR*_adj_ (%)		**−24.3**	−29.8	−43.8	**−30.5**	**−33.4**	**−44.5**		−16.6		**−40.3**			**−27.9**
Statistical confidence		**Yes**	No	No	**Yes**	**Yes**	**Yes**		No		**Yes**			**Yes**
		**October (Prevalent COVID in 2020)**			**November (Prevalent COVID in 2020)**				**December (Prevalent COVID in 2020)**					
		Sapporo	Tokyo	Osaka	Sapporo	Tokyo	Osaka		Sapporo		Tokyo			Osaka
2020 result	*MMT* (°C)	13.1	18.2	18.7	6.3	15.0	14.7		−1.6		9.0			8.7
	*MR*_adj_	10.11	10.50	9.42	10.10	11.20	11.31		10.71		12.75			11.35
*MMT* contribution rate from 10 years (%)		3.3	13.5	0.3	35.6	2.7	15.0		0.4		17.0			22.8
Expected natural *MR*_adj_		11.49	21.91	17.92	16.76	15.46	13.46		15.25		17.02			15.92
Predictable decrease of *MR*_adj_ (%)		**−34.3**	**−18.6**	**−22.5**	**−39.7**	−27.6	−16.0		**−29.8**		**−25.1**			**−28.7**
Statistical confidence		**Yes**	**Yes**	**Yes**	**Yes**	No	No		**Yes**		**Yes**			**Yes**

The “predictable change of *MR*_adj_” indicates the decrease rate (%) to “expected natural *MR*_adj_” estimated using a regression line determined by the past 10 year data. The bold numerals and characters in “predictable change of *MR*_adj_” and “statistical confidence” indicate a lower value than that at the 95% confidence interval determined from the past 10 years (shown as “Yes” in “statistical confidence”). In addition, the grey-coloured column corresponds to the city and month having a temperature-sensitive CI satisfying “*MMT* contribution rate from 10 years” greater than 10%.

Decreases in *MR*_adj_ satisfying both the *MMT* contribution rate exceeding 10% and the statistical confidence (coloured in grey in Tables 2 and 3) indicate that the mortality of diseases sensitive to temperature was significantly reduced due to the influence of the COVID-19 pandemic. On the other hand, the *MR*_adj_ of IHD and CI in the three months (January–March 2020) of pre-COVID-19 or spreading COVID demonstrated that meaningful decreases in *MR*_adj_ were not found in every city or month during those months compared with the past 10 years. This result suggests that the prevalence of COVID-19 has induced a decrease in cardiovascular disease mortality in Japanese society. Although Resp *MR*_adj_ already decreased with statistical significance in the months before April (cf. S1 Table), predictable decreases in *MR*_adj_ were smaller than those during the prevalent COVID-19 months.

**Fig 5:**
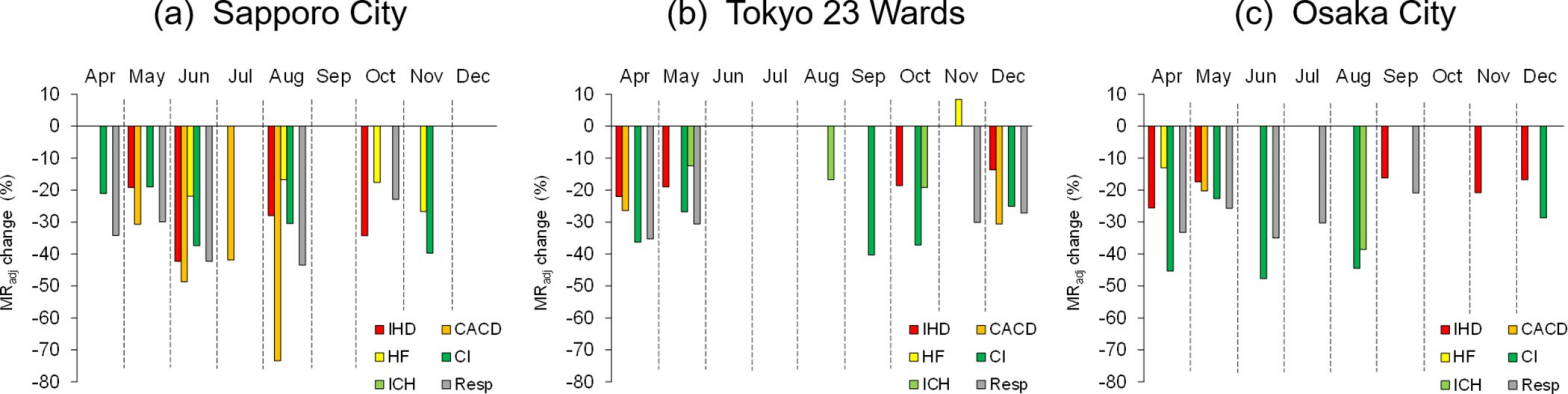
Statistically meaningful changes in *MR*_adj_ of each disease in the temperature-sensitive months of April–December 2020. The results of acute ischemic heart disease (IHD) (red), cardiac arrhythmia and conduction disorder (CACD) (orange), heart failure (HF) (yellow), cerebral infarction (CI) (green), intracerebral haemorrhage (ICH) (light green), and respiratory diseases (Resp) (grey) are provided in (a) Sapporo, (b) Tokyo, and (c) Osaka. Here, only temperature-sensitive months (as indicated in Tables 2 and 3) are evaluated in each disease.

Using these summaries (specified in the tables), a statistically significant change in the *MR*_adj_ of each disease was estimated for only temperature-sensitive months in the three cities during April–December 2020, when Japanese society suffered from the prevalence of COVID-19 (Fig 5). In Sapporo, which has the lowest temperature climate among the three cities (Fig 5A), decreases in CACD and Resp *MR*_adj_ were remarkable from spring to summer (April–August). A 73.4% reduction in the CACD *MR*_adj_ was found in August 2020, indicating that the CACD *MR*_adj_ could be increased by 3.8 times (1.49→5.60) for *MMT* of 23.3°C in 2020 if the COVID-19 spread had not occurred in Sapporo. In Tokyo (Fig 5B), the CI and Resp *MR*_adj_ especially decreased in the seasons other than summer. The CI *MR*_adj_ decreased by 40.3% in the temperature-sensitive month of September in Tokyo, which means that it could be increased by 1.7 times (9.00→15.08) for the *MMT* of 24.9°C in 2020 if the pandemic had not occurred. In Osaka (Fig 5C), decreases in CI, ICH, and Resp *MR*_adj_ appeared in the summer season, whereas the IHD *MR*_adj_ decreased in seasons other than summer. Among them, the CI *MR*_adj_ decreased by 47.7% in June in Osaka, meaning that it could have been increased by 1.9 times (8.98→17.15) for *MMT* of 24.9°C in 2020 if there were no COVID-19 pandemic.

### Increases of deaths if 2020 was unaffected by the COVID-19 pandemic

**Fig 6:**
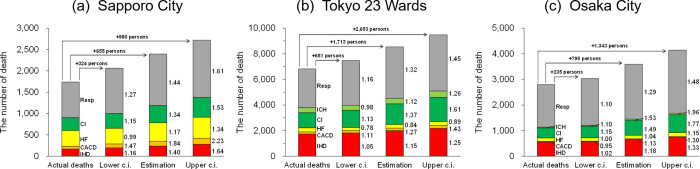
Estimated increases in death of each disease in April–December 2020 if the COVID-19 pandemic had not occurred. The results of acute ischemic heart disease (IHD) (red), cardiac arrhythmia and conduction disorder (CACD) (orange), heart failure (HF) (yellow), cerebral infarction (CI) (green), intracerebral haemorrhage (ICH) (light green), and respiratory diseases (Resp) (grey) are provided in (a) Sapporo, (b) Tokyo, and (c) Osaka. Here, only temperature-sensitive months (as indicated in Tables 2 and 3) are evaluated in each disease in the same manner as in Fig 5.

Based on the preceding results, the number of deaths, if unaffected by the COVID-19 pandemic, was quantitatively assessed from the actual *MMT* observed in 2020 (Fig 6). It should be noted that the increases in temperature-sensitive diseases and months were aggregated, and mortality in other diseases and months with no temperature response would also have increased if the pandemic has not occurred. In Sapporo (Fig 6A), 324–980 people from the 95% confidence interval (determined in Fig 4) were expected to increase the actual number of deaths from April to December in 2020. This corresponded to 1.19–1.56 times the deaths that were recorded. Deaths in Tokyo (Fig 6B) were expected to increase by a minimum of 651 people and a maximum of 2,653, which corresponded to 1.10–1.39 times the deaths actually recorded in April–December 2020. In Osaka (Fig 6C), the death increase of 235 to 1,343 people was estimated, corresponding to 1.08–1.48 times of the actual deaths recorded in the corresponding period. Consequently, death increases of 19–56%, 10–39%, and 8–48% were calculated in Sapporo, Tokyo, and Osaka, respectively, for temperature-sensitive diseases in Japan, if Japan had not experienced the pandemic in 2020. Among the temperature-sensitive diseases, deaths from Resp and CI indicated a higher increase rate common to the three cities, while higher increases in CACD and IHD deaths were found exclusively in Sapporo.

### Behaviour change in 2020

**Fig 7:**
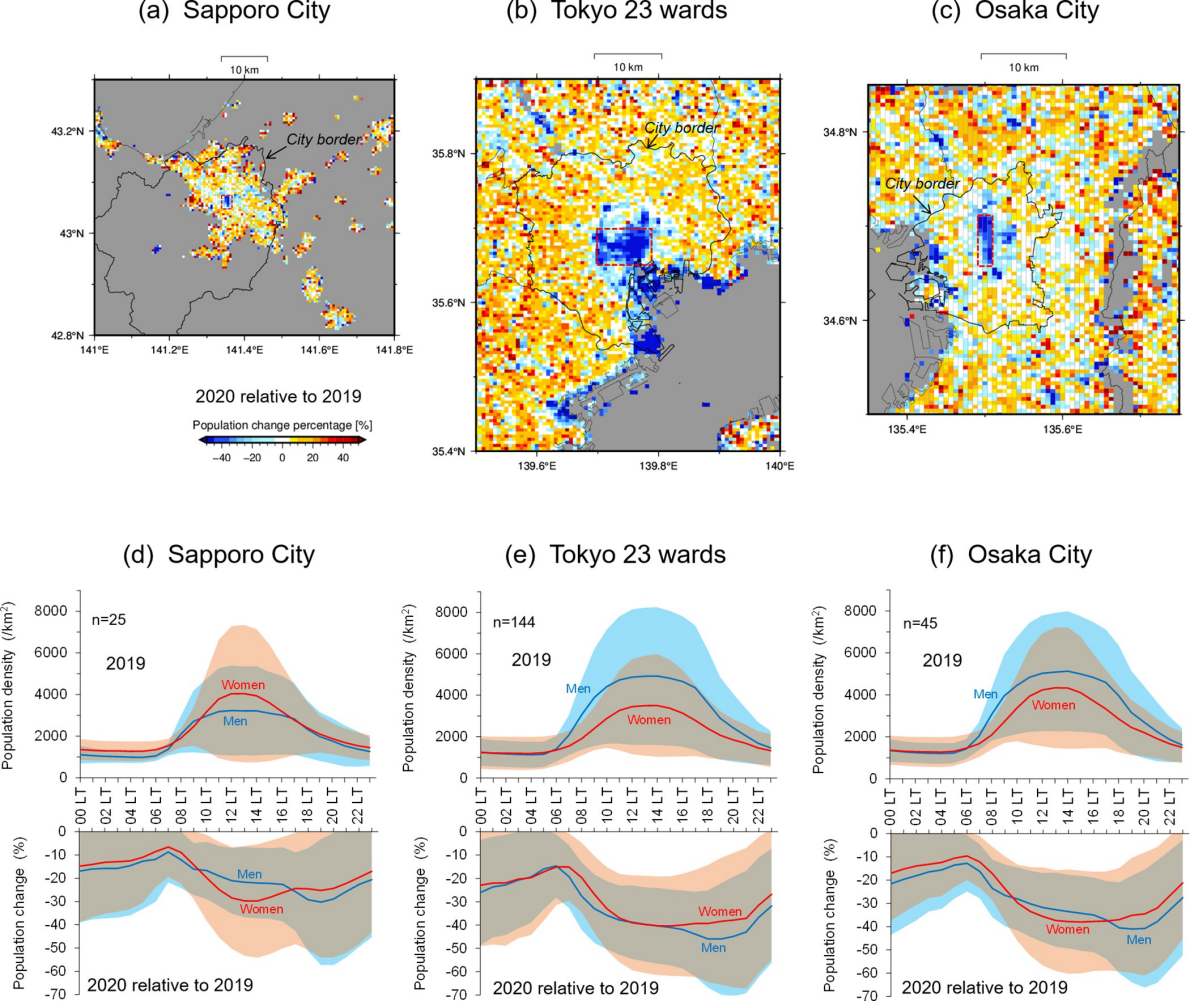
Elderly (60s and 70s) population change (%) averaged during an inhibited behaviour period in 2020 relative to 2019 at 12 LT. Spatial distributions obtained from mobile terminal information with a mesh of 1-km squares in (a) Sapporo, (b) Tokyo, and (c) Osaka. Red broken rectangles represent the central urban area analysed in (d)–(f). Hourly variations of population density per 1-km square at the upper panels of (d)–(f) and population change (%) in 2020 relative to 2019 at the lower panels of (d)–(f). Results averaged at the area depicted by the red broken rectangle in (a)–(c) (lines) and their standard deviations (tones) for men (blue) and women (red) in (d) Sapporo, (e) Tokyo, and (f) Osaka. These were averaged during an inhibited behaviour period from 17 April to 14 May in 2020.

Spatial distributions of change rate in population density from 2019 to 2020 during the state of emergency period from 17 April to 14 May, were analysed to assess mortality decreases (Fig 7A–7C). This map was produced by averaging the population of each mesh (1-km squares) of people in their 60s and 70s at noon on weekdays, which was acquired from the location information provided by mobile terminals (each population density map is shown in S2 Fig). Drastic population decreases occurred in the central district areas in every city; in particular, the phenomenon appeared in a broad area of approximately 10-km squares in the centre of Tokyo (Fig 7B). Because the population at 12 LT depicted in these maps was denser than those of morning or evening hours (upper panels in Fig 7D–7F), the maps when people were the most active are displayed in this figure.

At noon in the 2020 pandemic year, the Sapporo population of men in their 60s and 70s decreased by 21.1% as the area-average value, with a standard deviation (s.d.) of 14.1%, while that of women decreased by 28.5% (s.d. 21.9%) relative to 2019 (the lower part of Fig 7D). During the morning and night hours, excluding 10–17 LT, although the population decrease of older men was greater than that of women, the absolute population in the central areas of Sapporo was naturally lower in the corresponding hours (the upper part of Fig 7D). Calculating a daily population decrease in the central meshes as the average during an inhibited behaviour period, 12,781 and 16,235 older men and women, respectively, were decreased per mesh (1-km squares) in central areas of Sapporo. Similarly, the Tokyo population decreased by 38.8% (s.d. 15.8%) for older men and 39.0% (s.d. 20.3%) for older women at noon (lower part of Fig 7E) in the 2020 pandemic, while 27,950 and 17,126 people decreased daily for men and women, respectively, per central mesh. The Osaka older person population decreased by 31.8% (s.d. 14.9%) for men and 35.7% (s.d. 21.1%) for women at noon (lower part of Fig 7F), whereas 25,865 men and 20,844 women decreased daily per central mesh. The daily decrease in the ratio of men aged 60–79 to women was calculated as 0.79, 1.63, and 1.24 in Sapporo, Tokyo, and Osaka, respectively.

**Fig 8:**
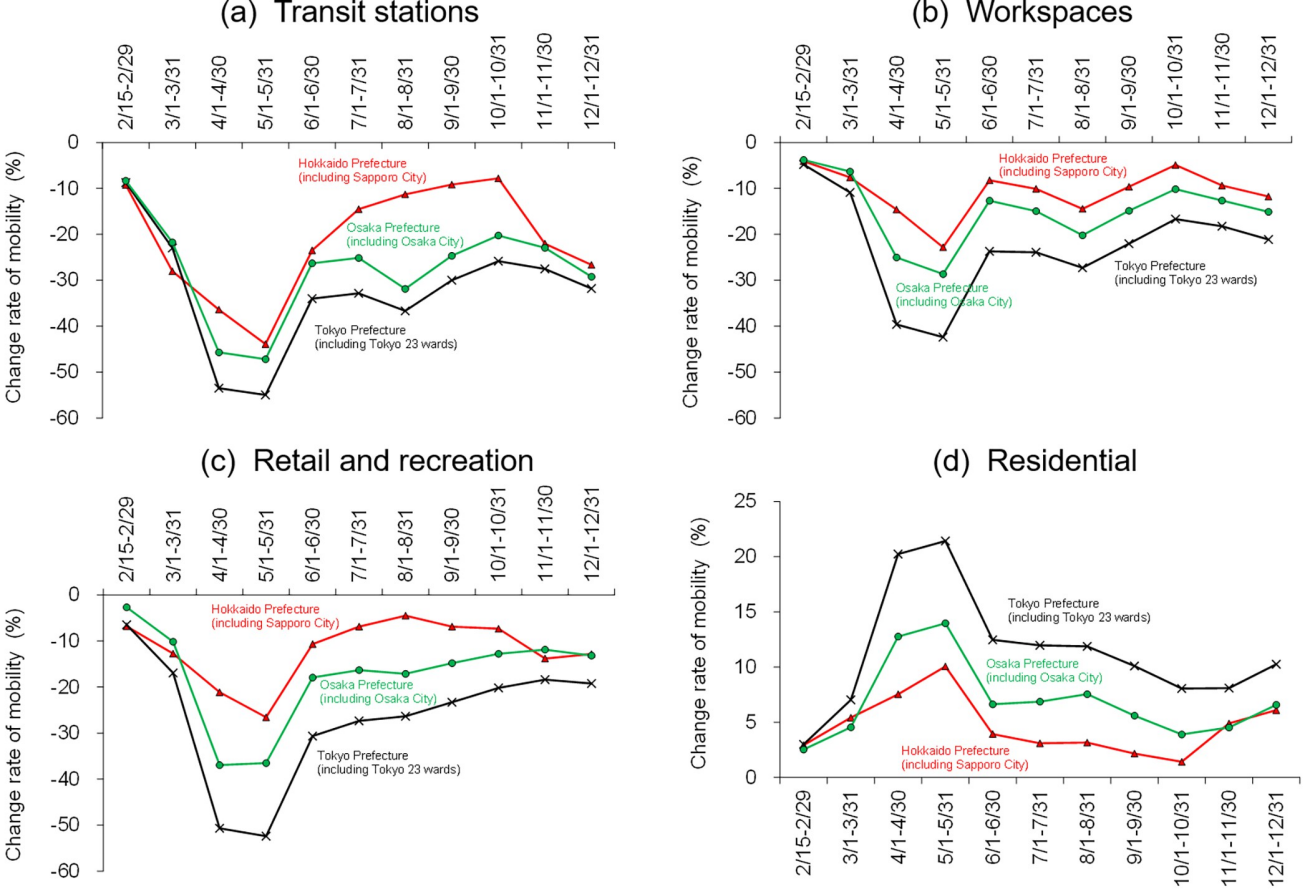
Temporal variations of the change rate of human mobility in Hokkaido prefecture (including Sapporo City), Tokyo prefecture (including Tokyo 23 wards), and Osaka prefecture (including Osaka City) during the period from 15 February to 31 December 2020. Results at (a) transit stations, (b) workspaces, (c) retail and recreation, and (d) residential, from the Google COVID-19 community mobility reports.

Unfortunately, in this study, the population mesh data from mobile terminals covering the broad areas shown in Fig 7 could not be acquired for 2020 before and after the inhibited behaviour period. However, the limited population data at stations and downtown meshes proved that the population decrease was still ongoing in 2020 after the termination of the state of emergency (cf. S3 Fig). In contrast, the daytime population increased in broad areas surrounding the central urban area (decreased in population) in every city compared with 2019 (Fig 7). This result suggests a city-scale behaviour change in which many people avoided unnecessary outings. The behaviour change phenomenon was already observed in March 2020, which can be confirmed by mobility changes from other information data; people mobility at stations, workspaces, and recreations in March 2020 decreased to be comparable to October and November 2020 at the prefecture scale, including in each city (Fig 8).

## Discussion

The number of deaths due to COVID-19 is reported independently of respiratory diseases in the Japanese government statistical data (https://www.e-stat.go.jp) from April to December 2020; 200, 537, and 248 deaths due to COVID-19 infection were recorded in Sapporo, Tokyo, and Osaka, respectively. The death increase estimates in a normal world unaffected by the pandemic estimated in this study were more than 10 times the number of COVID-19 deaths in each of the three cities. The incident risk of cardiovascular diseases due to COVID-19 infection has been reported by many researchers [33–36]. In the United States, IHD deaths significantly increased during the COVID-19 pandemic compared with the previous year [37]. Moreover, in England and Wales, the excess mortality from cardiovascular diseases was estimated from direct effects due to infection and indirect effects due to unprecedented system strain and associated behavioural changes [38]. In addition, heat-related mortality in Portugal in 2020 has been indirectly amplified by the COVID-19 pandemic due to the disruption of healthcare systems and fear of the population attending healthcare facilities [39]. These results likely contradict the results of our study, which used Japanese mortality data.

Some studies have reported that the nationwide lockdown due to COVID-19 increased mortality from cardiovascular diseases [40, 41] and decreased mortality from respiratory diseases [42]. The results obtained in the current study clarified that decreasing the hours of total exposure to outdoor temperatures reduced the mortality risk of temperature-sensitive diseases.

**Fig 9:**
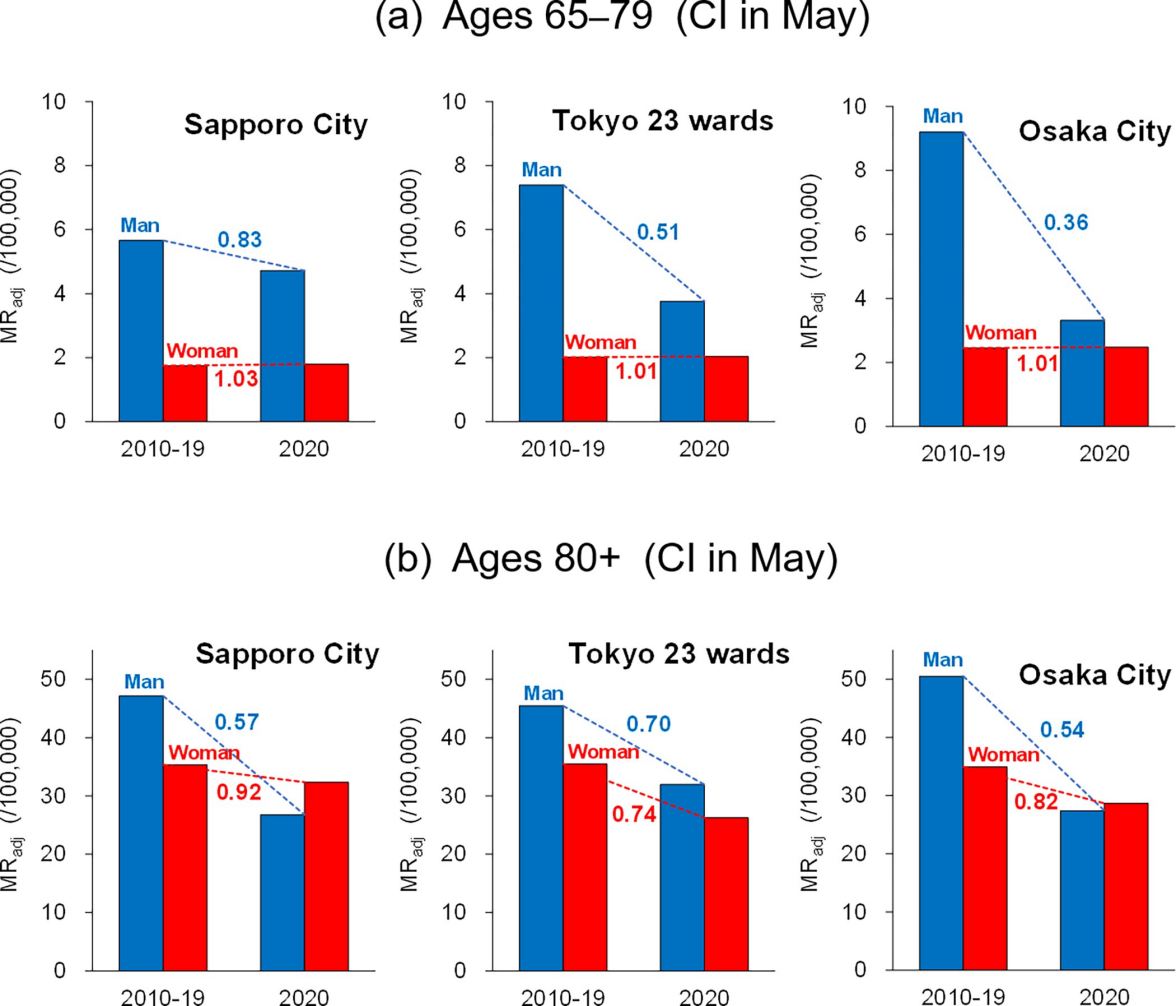
Comparison of the cerebral infarction (CI) *MR*_adj_ in older men and women between 2010–2019 and 2020. The results of (a) 65–79 age group and (b) 80 and older age group are displayed for May, in which temperature-sensitive CI was found in all cities. Numerals for men and women are the ratio of the 2020 *MR*_adj_ to the 2010–2019 averaged *MR*_adj_.

Based on the abovementioned behaviour change, differences in the *MR*_adj_ between men and women aged 65–79 that were retrieved by the previous population change were additionally analysed in Fig 9A for the CI in May, where the *MR*_adj_ decreased with temperature sensitivity in all cities. In this age group, women’s *MR*_adj_ was significantly lower than that of men and was almost unchanged under the 2020 pandemic; that is, the decrease in men’s *MR*_adj_ affected the overall decline, especially in Tokyo (49% decrease for averaging 2010–2019) and Osaka (64% decrease). This result is consistent with the greater decrease in the male population in 2020 in Tokyo and Osaka (Fig 7E and 7F). On the other hand, *MR*_adj_ did not decrease in the 65–79 age group despite a decrease in the female population in 2020 (women decreased more than men in Sapporo). As shown in Fig 9A, women’s *MR*_adj_ for cardiovascular diseases was naturally low in the corresponding age group. This may be attributed to various factors including lifestyle differences and the influence of sex hormones [43–45].

Mobile terminal data used in this study for people aged ≥80 were not provided because of the small sample size. As indicated in Fig 9B, inhibited behaviour in this age group may have induced a decrease in *MR*_adj_ not only in men (in three cities, 30–46% decrease on average 2010–2019) but also in women (8–26% decrease in three cities). In this age group, the women’s *MR*_adj_ for cardiovascular diseases tended to naturally increase, unlike the 60s and 70s age groups.

The results revealed by the current study indicate that temperature changes exposed in daily life affect human deaths due to cardiovascular and respiratory diseases and are also helpful in considering individual risk management for health. In particular, older people at high risk for these diseases should avoid the risk of cumulatively increasing their livelihood in temperature-controlled indoor spaces in the shortly term. However, it should be noted that this study’s conclusion does not apply to long-term influences over several years or decades.

Currently, the Japanese death data for 2021 are published as a preliminary report from the government. For example, the results for May 2021 indicated that the IHD *MR*_adj_ in Sapporo, Tokyo, and Osaka increased from 8.44, 15.87, and 12.29 to 9.86, 17.93, and 13.90, respectively. These returned to *MR*_adj_ in the pre-COVID-19 inferred from *MMT* (13.1°C in Sapporo, 19.8°C in Tokyo, and 20.0°C in Osaka) observed in May 2021. In fact, a significant recovery of human activities was confirmed in station and downtown areas (25–135% increase in May 2021 to the same month of 2020). However, it should be noted that preliminary mortality values are tentative information often corrected in the final report.

## Conclusion

This study clarified mortality changes associated with temperature-sensitive cardiovascular and respiratory diseases in Japan during the COVID-19 pandemic using data from three major cities (Sapporo City, Tokyo 23 wards, and Osaka City) from 2010–2020 to analyse relationship between disease mortality rates and monthly mean temperatures. A comparison between the regression results obtained from the past 2010–2019 and those in 2020 described the impact of the COVID-19 pandemic society on changes in temperature-sensitive disease mortality in Japan. If the COVID-19 pandemic had not occurred in 2020, temperature-sensitive disease death counts would have significantly increase from 324 to 980 people, based on a 95% confidence interval estimated from the past 10 years in Sapporo (19–56% increase in actual deaths from 2020), from 651 to 2,653 in Tokyo (10–39% increase), and from 235 to 1,343 in Osaka (8–48% increase) due to temperature changes in people’s livelihoods.

Heat exposure enhances dehydration of the human body, while cold exposure increases blood pressure due to the vasoconstriction response and increases blood viscosity [46, 47]. Moreover, a significant change in air temperature surrounding the human body induces blood pressure and heart rate instability [48]. For example, these physiological responses increase the risk of thrombosis (ischaemic heart disease and cerebral infarction) and blood vessel rupture (intracerebral haemorrhage). These facts suggest that acute incidences of temperature-sensitive diseases, such as cardiovascular and respiratory diseases, can be controlled by avoiding exposure to extreme temperatures or their changes. Such knowledge and understanding benefit health management and can alert those diseases risk for older people vulnerable to heat or cold, for example, to make an effort to increase air-conditioned indoor activities when it is hotter or colder than usual outdoors.

However, long-term effects of the COVID-19 pandemic on human health remain unclear. For example, many months of staying at home may lead to health problems such as decreasing physical activity, and may cause a negative effect on cardiovascular and respiratory diseases. Hence, based on both short-term and long-term risks, the actual reasons and pathogenesis on mortality changes of temperature-sensitive diseases need to be clarified in future works.

## Supporting information

S1 Fig The *MR*_adj_ sensitivity to *MMT* analysed from the past data and the 2020 plots in Sapporo (red triangle), Tokyo (black cross), and Osaka (green circle). (PDF)

S2 Fig Population densities of older (60s and 70s) men, women, and both total in (a) Sapporo City, (b) Tokyo 23 wards, and (c) Osaka City.
(PDF)

S3 Fig Daily variations of decrease rate of population in 2020 to a same month in 2019 of pre-pandemic years at a station and downtown areas in Sapporo, Tokyo, and Osaka. (PDF)

S1 Table Predictable changes in *MR*_adj_ for respiratory diseases (Resp) estimated in this study. (PDF)

S1 Data Dataset of MMT and *MR*_adj_. (XLSX)
