# Supplementary material for: Impact of the COVID-19 pandemic on changes in temperature-sensitive cardiovascular and respiratory disease mortality in Japan
Source: PLoS One. 2022 Oct 10;17(10):e0275935. doi: 10.1371/journal.pone.0275935 (PMC9550070; doi:10.1371/journal.pone.0275935)
Supplement: S1 Fig — (PDF) [file pone.0275935.s001.pdf]

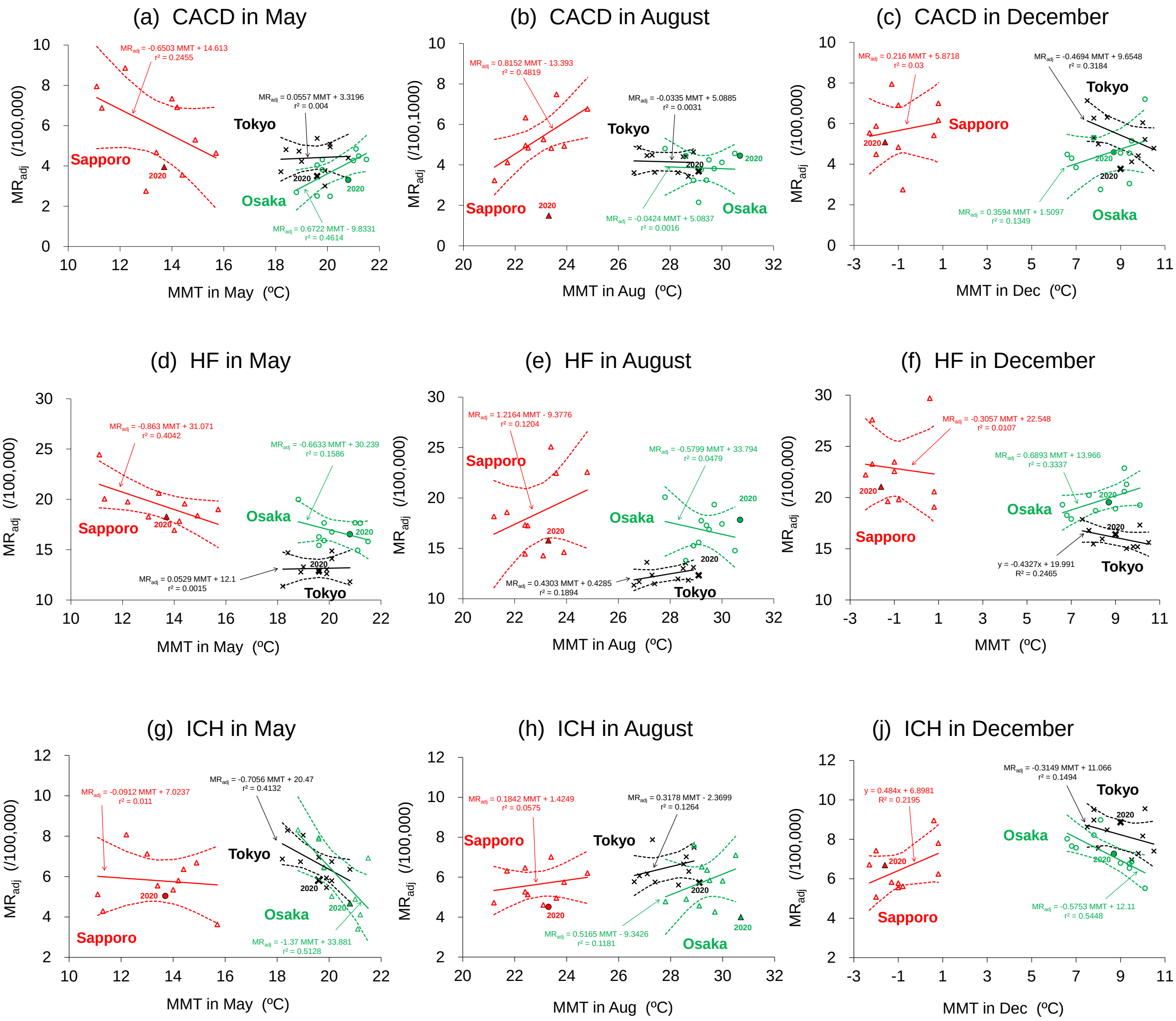

The results of (a, b, c) cardiac arrhythmia and conduction disorder (CACD), heart failure (HF) (d, e, f), and intracerebral haemorrhage (ICH) (g, h, i) are exhibited for May, August, and December. Straight regression line and the 95% confidence interval (broken curve line) are presented for the pre-pandemic (2010–2019), while filled marks represent the results of the COVID-19 pandemic year (2020). Regression equations and their contribution rates are also shown for the regression lines.
