# Supplementary material for: Impact of the COVID-19 pandemic on changes in temperature-sensitive cardiovascular and respiratory disease mortality in Japan
Source: PLoS One. 2022 Oct 10;17(10):e0275935. doi: 10.1371/journal.pone.0275935 (PMC9550070; doi:10.1371/journal.pone.0275935)
Supplement: S2 Fig — Population densities of older (60s and 70s) men, women, and both total in (a) Sapporo City, (b) Tokyo 23 wards, and (c) Osaka City. (PDF) [file pone.0275935.s002.pdf]

(a)

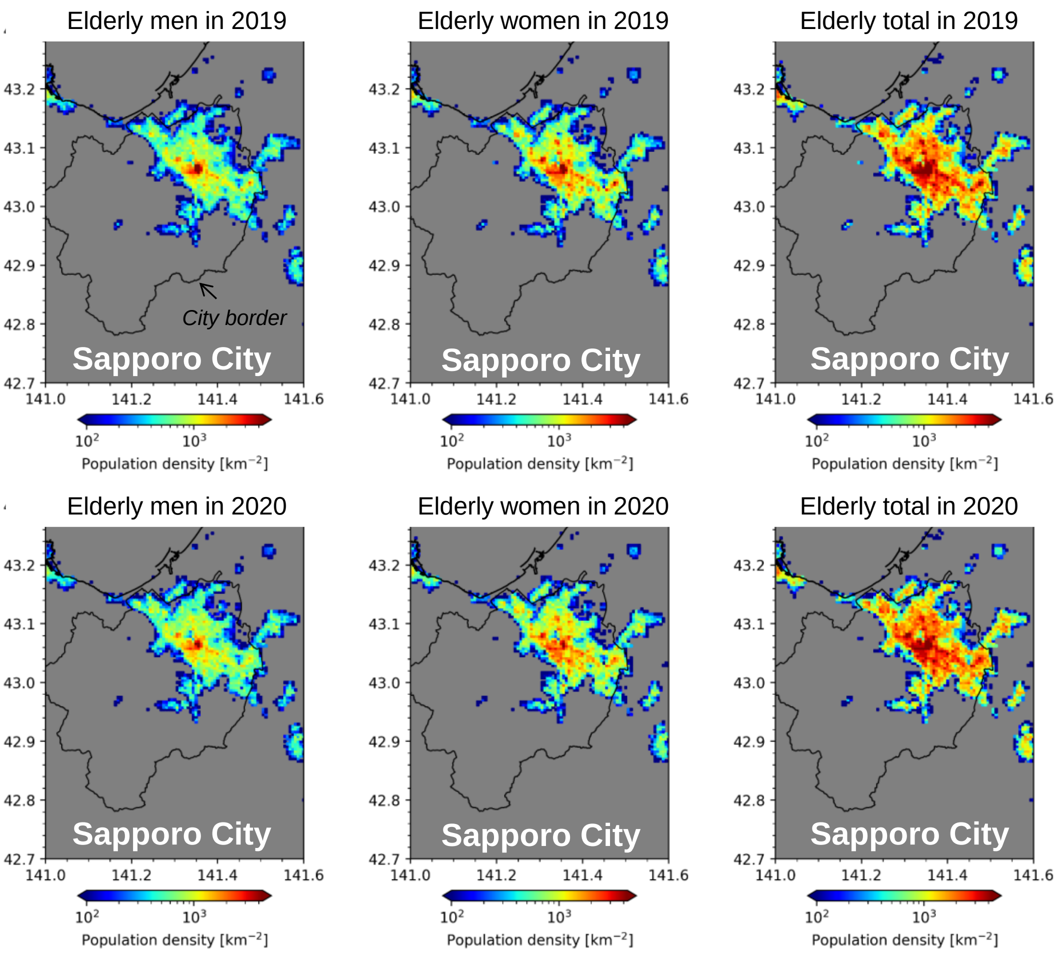
Elderly men in 2019
Elderly women in 2019
Elderly total in 2019
Elderly men in 2020
Elderly women in 2020
Elderly total in 2020
City border
Sapporo City
43.2
43.1
43.0
42.9
42.8
42.7
141.0
141.2
141.4
141.6
10^2
10^3
Population density [km^{-2}]

(b)

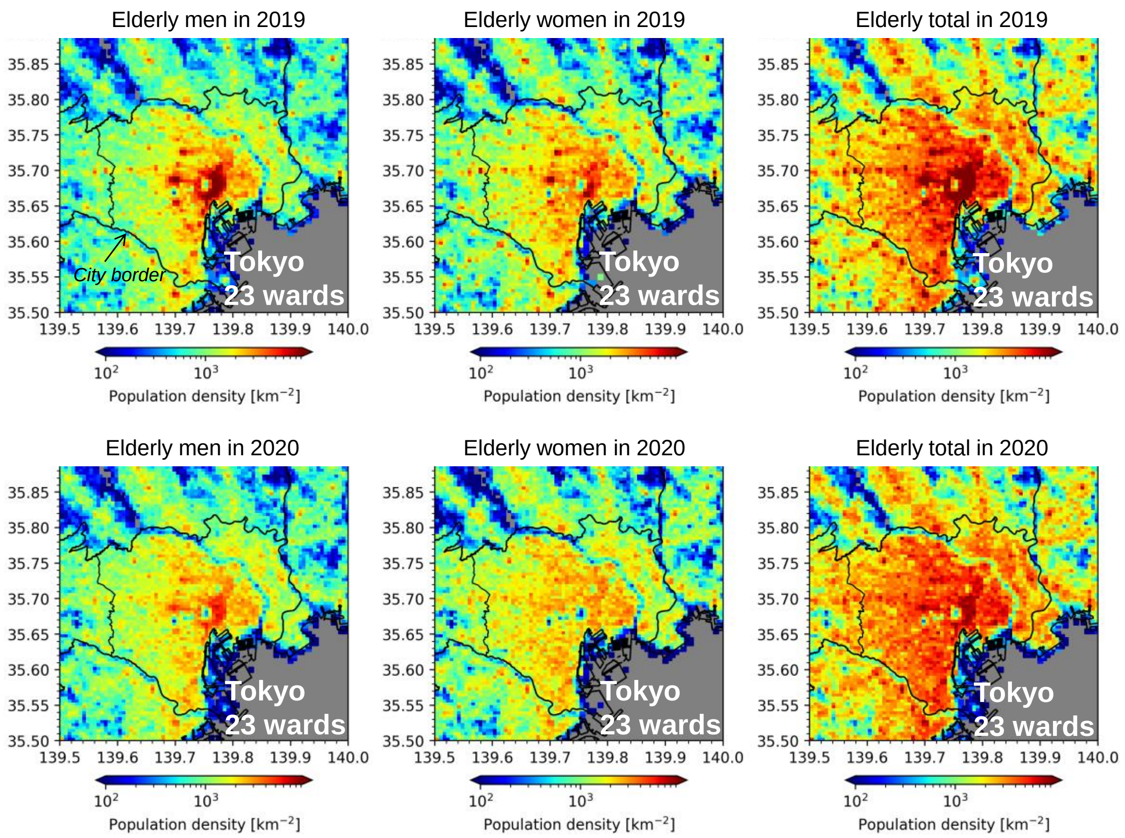
Elderly men in 2019
Elderly women in 2019
Elderly total in 2019
Elderly men in 2020
Elderly women in 2020
Elderly total in 2020
City border
Tokyo
23 wards
35.85
35.80
35.75
35.70
35.65
35.60
35.55
35.50
139.5
139.6
139.7
139.8
139.9
140.0
10^2
10^3
Population density [km^{-2}]

(c)

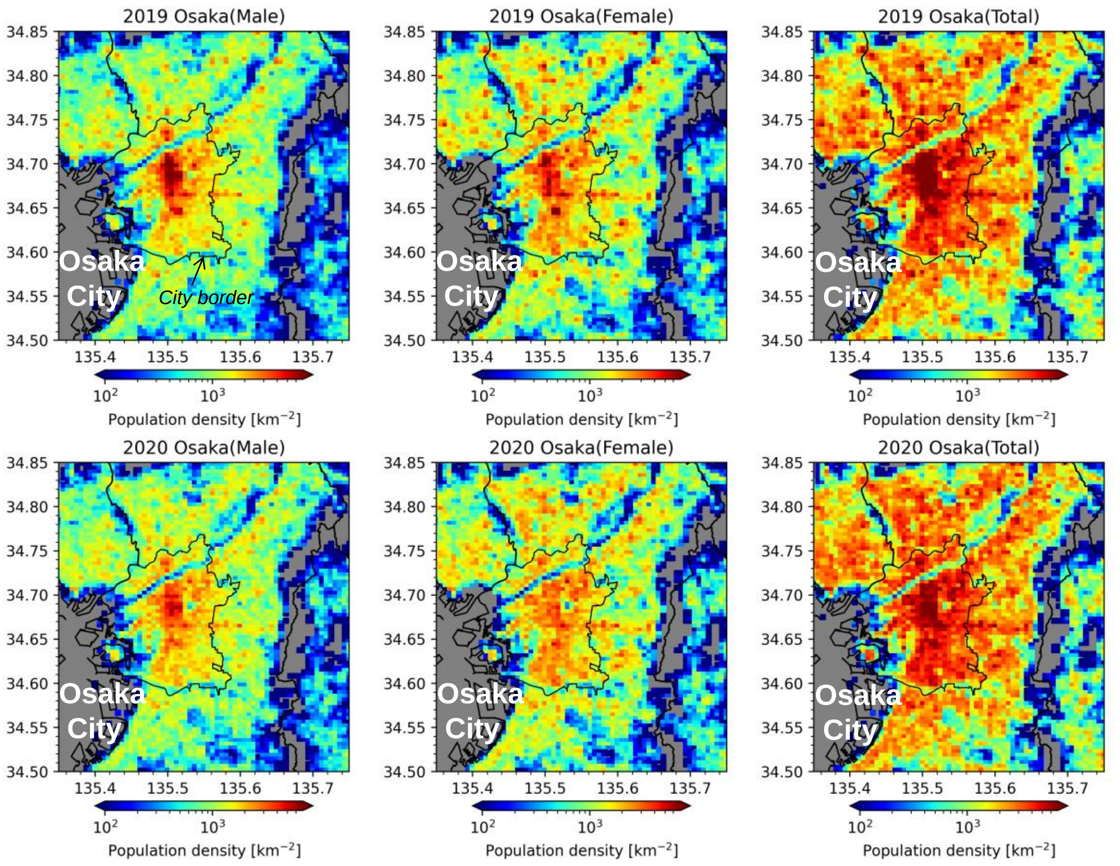

Results averaged from 17 April to 14 May at 12 LT in 2019 of the pre-COVID normal year (upper panel) and 2020 of the COVID-19 pandemic year (lower panel).
