# Supplementary material for: Impact of the COVID-19 pandemic on changes in temperature-sensitive cardiovascular and respiratory disease mortality in Japan
Source: PLoS One. 2022 Oct 10;17(10):e0275935. doi: 10.1371/journal.pone.0275935 (PMC9550070; doi:10.1371/journal.pone.0275935)
Supplement: S3 Fig — (PDF) [file pone.0275935.s003.pdf]

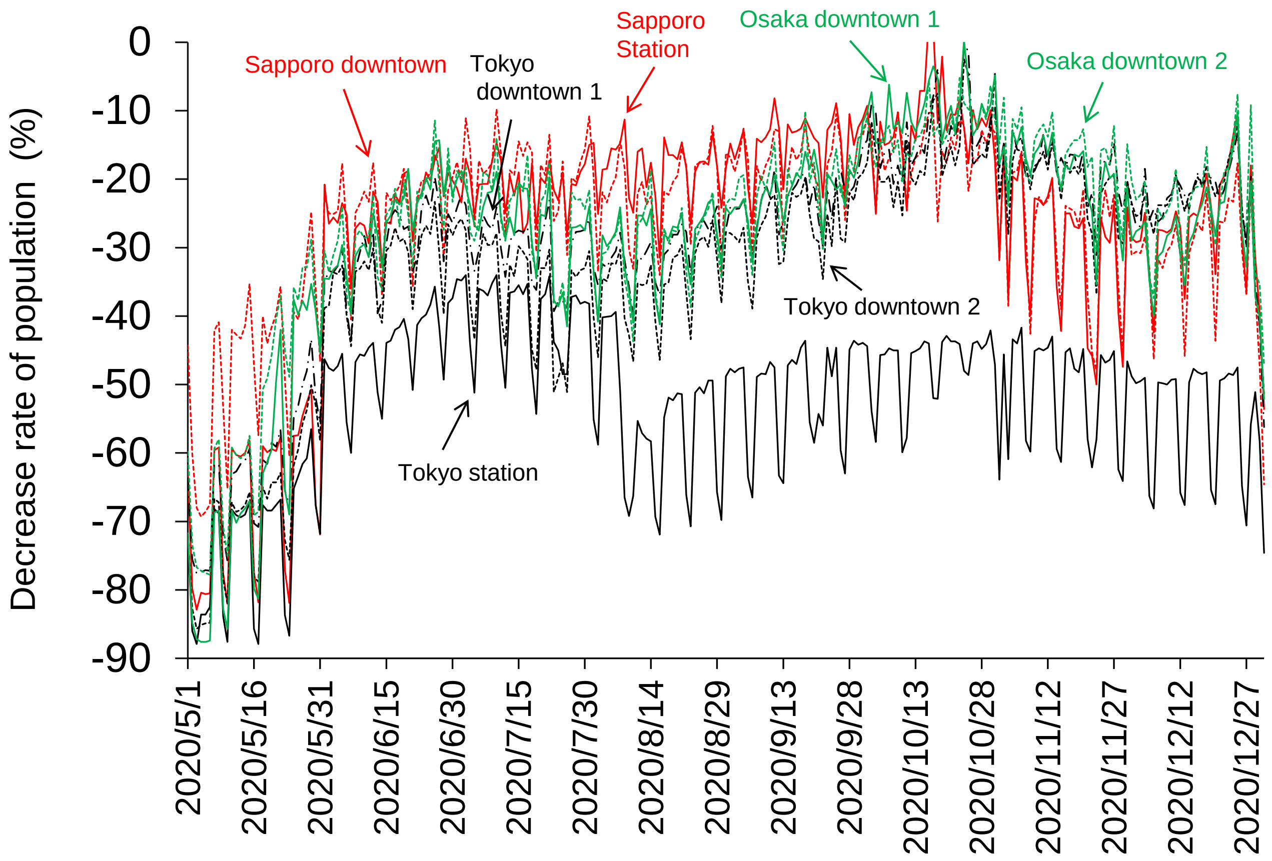

The analyses at 15 LT during the period of 1 May to 31 Dec are exhibited in this figure. Population decrease rates at station and downtown areas indicate the change for one week and showed a larger decrease rate of weekend in the all areas.
