# Supplementary material for: Impact of the COVID-19 pandemic on changes in temperature-sensitive cardiovascular and respiratory disease mortality in Japan
Source: PLoS One. 2022 Oct 10;17(10):e0275935. doi: 10.1371/journal.pone.0275935 (PMC9550070; doi:10.1371/journal.pone.0275935)
Supplement: S1 Table — (PDF) [file pone.0275935.s004.pdf]

		**January (Pre-COVID in 2020)**			**February (Pre-COVID in 2020)**			**March (Spreading COVID in 2020)**			**April (Prevalent COVID in 2020)**			**May (Prevalent COVID in 2020)**			**June (Prevalent COVID in 2020)**		
		Sapporo	Tokyo	Osaka	Sapporo	Tokyo	Osaka	Sapporo	Tokyo	Osaka	Sapporo	Tokyo	Osaka	Sapporo	Tokyo	Osaka	Sapporo	Tokyo	Osaka
2020 result	*MMT* (°C)	−2.3	8.2	8.6	−2.1	9.1	8.0	3.3	11.4	11.4	6.8	13.4	13.7	13.7	19.6	20.8	18.3	23.3	24.9
	MR_{adj}	40.18	44.62	57.92	31.63	37.68	54.09	34.34	35.64	51.55	28.18	33.58	45.23	29.47	30.94	42.08	27.9	27.93	40.41
MMT contribution rate from 10-yrs (%)		8.4	24.5	16.9	12.0	37.7	11.7	22.5	19.6	7.2	23.9	35.1	36.2	39.6	51.8	57.7	51.2	0.9	15.2
Expected natural MR_{adj}		51.82	53.88	62.43	44.53	44.63	63.97	41.01	48.41	63.35	42.81	51.88	67.84	42.05	44.57	56.67	48.39	39.20	62.14
Predictable change of MR_{adj} (%)		**−22.5**	−17.2	−7.2	**−29.0**	−15.6	**−15.4**	**−16.3**	**−26.4**	**−18.6**	**−34.2**	**−35.3**	**−33.3**	**−29.9**	**−30.6**	**−25.7**	**−42.3**	−28.8	**−35.0**
Statistical confidence		**Yes**	No	No	**Yes**	No	**Yes**	**Yes**	**Yes**	**Yes**	**Yes**	**Yes**	**Yes**	**Yes**	**Yes**	**Yes**	**Yes**	No	**Yes**

MMT: monthly mean temperature.

MR_{adj}: monthly mortality rate per 100,000 people aged 65 and older.

		July (Prevalent COVID in 2020)			August (Prevalent COVID in 2020)			September (Prevalent COVID in 2020)			October (Prevalent COVID in 2020)			November (Prevalent COVID in 2020)			December (Prevalent COVID in 2020)		
		Sapporo	Tokyo	Osaka	Sapporo	Tokyo	Osaka	Sapporo	Tokyo	Osaka	Sapporo	Tokyo	Osaka	Sapporo	Tokyo	Osaka	Sapporo	Tokyo	Osaka
2020 result	*MMT* (°C)	21.2	24.7	26.0	23.3	29.1	30.7	20.1	24.9	25.8	13.1	18.2	18.7	6.3	15.0	14.7	−1.6	9.0	8.7
	MR_{adj}	27.72	30.49	44.14	25.9	31.66	46.52	30.18	32.91	42.64	33.1	35.09	48.97	31.62	32.96	43.87	34.64	39.26	50.47
MMT contribution rate from 10-yrs (%)		0.2	5.1	14.6	53.9	2.0	8.2	3.9	1.80	13.8	13.4	4.6	1.2	1.2	12.8	7.7	1.1	12.2	2.2
Expected natural MR_{adj}		43.33	40.23	63.29	45.83	46.32	61.19	45.21	44.30	53.89	42.94	49.59	57.04	47.05	47.18	58.84	46.45	53.96	66.65
Predictable decrease of MR_{adj} (%)		**−36.0**	**−24.2**	**−30.3**	**−43.5**	**−31.7**	**−24.0**	**−33.2**	**−25.7**	**−20.9**	**−22.9**	**−29.2**	−14.2	**−32.8**	**−30.1**	**−25.4**	**−25.4**	**−27.2**	**−24.3**
Statistical confidence		**Yes**	**Yes**	**Yes**	**Yes**	**Yes**	**Yes**	**Yes**	**Yes**	**Yes**	**Yes**	**Yes**	No	**Yes**	**Yes**	**Yes**	**Yes**	**Yes**	**Yes**

The "predictable change of MR_{adj}" indicates the decrease rate (%) to "expected natural MR_{adj}" estimated using a regression line which is determined by the past 10-year data. The bold numerals and characters in "predictable change of MR_{adj}" and "statistical confidence" indicate the lower value than that at the 95% confidence interval determined from the past 10 years (shown as "Yes" in "statistical confidence"). In addition, the grey-coloured column corresponds to the city and month having the temperature-sensitive Resp satisfying "*MMT* contribution rate from 10-yrs" greater than 10% of them.
